# Quantitative Imaging in Oncology

**DOI:** 10.3390/tomography8040139

**Published:** 2022-06-24

**Authors:** Wonmo Sung

**Affiliations:** Department of Biomedical Engineering and of Biomedicine & Health Sciences, College of Medicine, The Catholic University of Korea, Seoul 06591, Korea; wsung@catholic.ac.kr

The Special Issue of *Tomography* is a collection of articles focused on the quantitative imaging methods in clinical oncology.

Cancer is a leading cause of death. The gene mutation in cancer cells accelerates cell division or inhibits normal controls. As the tumor grows rapidly, it can disturb proper organ function. If vital organ function is impaired, it can lead to patient death. The use of imaging techniques allows doctors to pinpoint the tumor mass in the three-dimensional space of the human body.

Modern imaging techniques transform medicine from art to quantitative science. The energy wave is used to penetrate, classify, and locate the substance properties in the body. For example, the developments in computed tomography (CT), mammography, magnetic resonance imaging (MRI), and ultrasound result in quantitative anatomical evaluation in medicine. Moreover, the use of radiotracers enables functional imaging to be performed, such as positron emission tomography (PET) and single-photon emission computed tomography (SPECT), in nuclear medicine. These imaging techniques have converted a qualitatively ambiguous disease into quantitative numbers. This has become particularly important in cancer treatment.

Quantitative imaging is mainly used for three purposes in oncology: classification, detection, and segmentation. (1) Image classification allows us to classify what is contained in an image. Park et al. evaluated MR radiomic features as a prognostic factor for predicting ipsilateral recurrence of DCISs [1]. (2) Object detection specifies the quantitative objects in the image. Li et al. analyzed the quantitative changes in the airway in the chest CT of patients with fibrotic interstitial lung abnormalities [2]. (3) Image segmentation creates a pixel-wise mask of each object in the images. For example, creating a mask indicating the tumor location is an important process to maximize the radiation damage to the tumor while reducing the radiation damage to the normal tissues [3]. These three goals of quantitative imaging are fast-evolving fields, which are being aided by recent advances in deep learning.

Quantitative imaging can also be used for monitoring therapeutic efficacy, tumor progression, and guiding therapeutic and diagnostic interventions. Radke et al. introduced a chemical exchange saturation transfer imaging method for lactate-weighted MRI over time [4]. The modeling of time-series imaging will also enable personalized adaptive chemo- and radiotherapy to be performed, ultimately improving the survival of patients with cancer [5].

In conclusion, this Special Issue collects recent research papers to assess cancer diagnosis and therapy, aided by the above quantitative imaging techniques. Our investigations should help to better assess cancer risk and help patients with cancer to improve their health and survival.
